# The Pharmacological Activities of the Metabolites of N-[(Trimethylamineboryl)-Carbonyl]-L-Phenylalanine Methyl Ester

**DOI:** 10.1155/MBD.1996.219

**Published:** 1996

**Authors:** M. C. Miller, A. Sood, B. F. Spielvogel, R. P. Shrewsbury, I. H. Hall

**Affiliations:** 1 Division of Medicinal Chemistry & Natural Products School of Pharmacy University of North Carolina Chapel Hill NC 27599-7360 USA; 2 Boron Biologicals Inc. 620 Hutton St. Raleigh NC 27606 USA; 3 Division of Pharmaceutics School of Pharmacy University of North Carolina Chapel Hill NC 27599-7360 USA

## Abstract

The metabolites of N-[(trimethylamineboryl)-carbonyl]-L-phenylalanine methyl ester **1** proved to be active in a number of pharmacological screens where the parent had previously demonstrated potent activity. The proposed metabolites demonstrated significant activity as cytotoxic, hypolipidemic, and anti-inflammatory agents. In cytotoxicity screens several of the proposed metabolites afforded better activity than the parent compound against the growth of suspended and solid tumor cell lines. Evaluation of *in vivo* hypolipidemic activity demonstrated that the proposed metabolites of **1** were only moderately active and were generally less effective than the parent compound. Interestingly, L-phenylalanine methyl ester hydrochloride **3**, which contains no boron atom, demonstrated equivalent hypolipidemic activity as the parent at **8** mg/kg/day in CF_1_ male mice. As anti-inflammatory agents the proposed metabolites demonstrated variable capacities to reduce foot pad inflammation. These compounds were similarly effective as the parent **1** at blocking local pain and were generally better than the parent at protecting CF_1_ male mice from LPS induced sepsis.


### THE PHARMACOLOGICAL ACTIVITIES OF THE METABOLITES OF N-[(TRIMETHYLAMINEBORYL)-CARBONYL]-L-PHENYLALANINE METHYL ESTER

M. C. Miller, III,[1] A. Sood,[2] B. F. Spielvogel,[2] R. P. Shrewsbury,[3] and I. H. Hall*[1]
[1] Division of Medicinal Chemistry & Natural Products
and [3] Division of Pharmaceutics,
School of Pharmacy, University of North Carolina, Chapel Hill, NC 27599-7360, USA
[2] Boron Biologicals, Inc., 620 Hutton St., Raleigh, NC 27606, USA

Abstract:

The metabolites of N-[(trimethylamineboryl)-carbonyl]-L-phenylalanine methyl ester **1** proved to be active in a number of pharmacological screens where the parent had previously demonstrated potent activity. The proposed metabolites demonstrated significant activity as cytotoxic, hypolipidemic, and anti-inflammatory agents. In cytotoxicity screens several of the proposed metabolites afforded better activity than the parent compound against the growth of suspended and solid tumor cell lines. Evaluation of *in vivo* hypolipidemic activity demonstrated that the proposed metabolites of **1** were only moderately active and were generally less effective than the parent compound. Interestingly, L-phenylalanine methyl ester hydrochloride **3**, which contains no boron atom, demonstrated equivalent hypolipidemic activity as the parent at 8 mg/kg/day in $CF_1$ male mice. As anti-inflammatory agents the proposed metabolites demonstrated variable capacities to reduce foot pad inflammation. These compounds were similarly effective as the parent **1** at blocking local pain and were generally better than the parent at protecting $CF_1$ male mice from LPS induced sepsis.

Introduction:

N-[(Trimethylamineboryl)-carbonyl]-L-phenylalanine methyl ester **1** has been shown to possess antitumor, hypolipidemic, and antiinflammatory activities *in vivo* [1]. Disposition and distribution studies of this compound in $CF_1$ male mice after intraperitoneal or intravenous administration indicated it was rapidly metabolized to N-[(trimethylamineboryl)-carbonyl]-L-phenylalanine in the plasma. In simulated gastric fluid, compound **1** was rapidly hydrolyzed to L-phenylalanine methyl ester **3** [2]. In order to address concerns that metabolites may be responsible for the pharmacological activity, synthesis and pharmacological evaluation of possible metabolic products of compound **1** were undertaken. The metabolic products evaluated included sodium N-[(trimethylamineboryl)-carbonyl]-L-phenylalanine **2**, L-phenylalanine methyl ester **3**, L-phenylalanine **4**, and boric acid **5**. Additionally, a non-boronated derivative, N,N-dimethylglycyl-L-phenylalanine methyl ester **6**, was evaluated to determine the necessity of the boron atom for pharmacological activity (Figure I).

Materials and Methods:

Chemistry:

Reagents and Apparatus:

All chemicals were used as received from the manufacturer. Solvents were distilled prior to use. Trimethylamine carboxyborane was provided by Boron Biologicals, Inc. (Raleigh, NC).
All other chemicals used in the synthetic procedures were purchased from Aldrich Chemical Company (Milwaukee, WI). A Perkin Elmer 1320 Infrared Spectrophotometer was used for infrared (IR) analyses using KBr disks or nujol mulls between sodium chloride plates. A Varian 300 MHz NMR spectrometer was used to generate $^1$H-NMR spectra. Chemical shifts are relative to the external standard tetramethylsilane ($\delta$ = 0). A Thomas-Hoover capillary melting point apparatus was used to determine melting points, which were uncorrected. Elemental analyses were performed by M-H-W Laboratories (Phoenix, AZ). Silica gel 60F 254 plates (silica gel on aluminum, Aldrich Chemical Company) were used for thin layer chromatography.

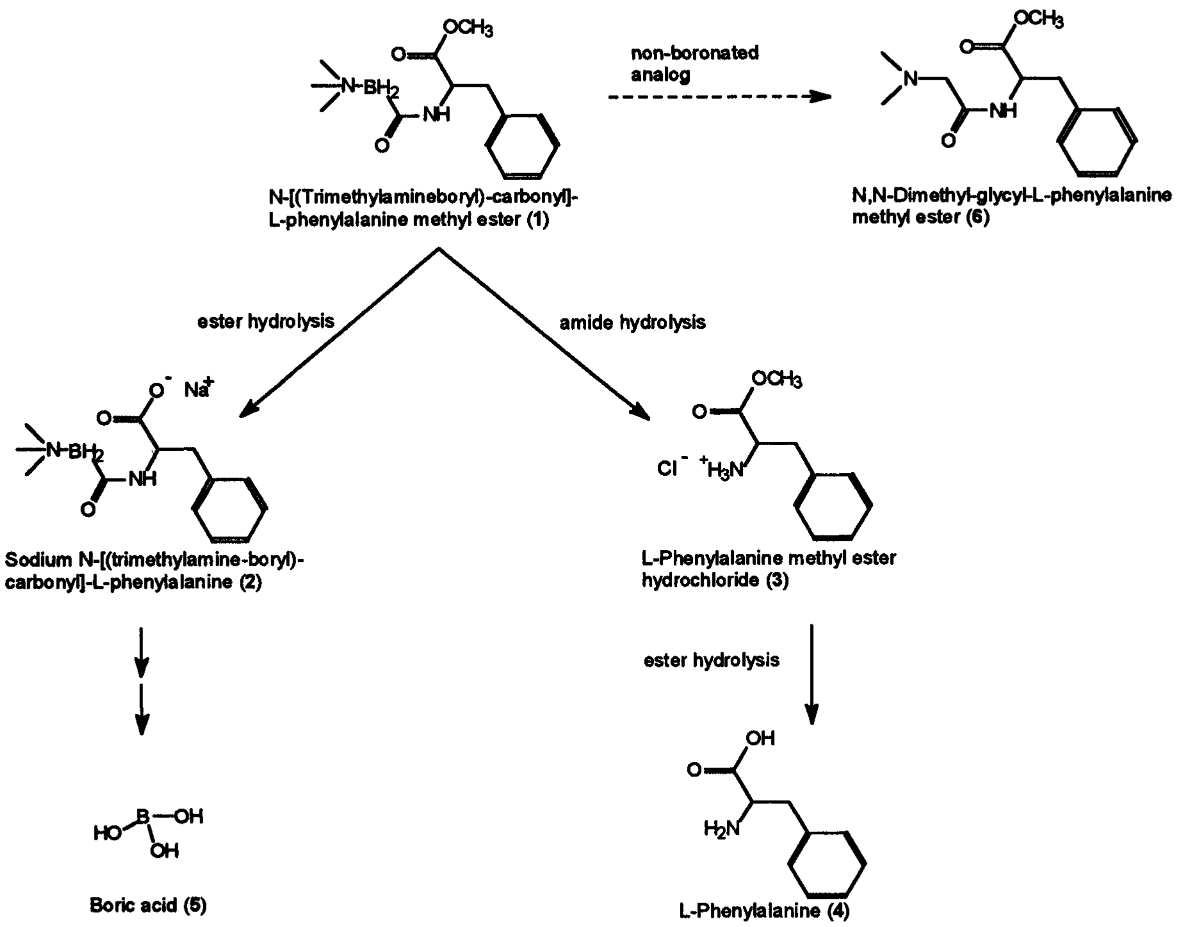

**Figure I: Molecular Structures of the Derivatives of the Boronated Dipeptide N-[(Trimethylamineboryl)-carbonyl]-L-phenylalanine Methyl Ester.**

Synthesis:

The synthesis of boronated dipeptides was accomplished by using the coupling agent triphenylphosphine/carbon tetrachloride (Scheme I). The conditions under which this reaction was conducted were mild enough so that the yields were of acceptable levels compared to traditional condensation conditions, such as thionyl chloride, that result in the formation of chloroborane instead of the desired product [3]. The mechanism of $PPh_3/CCl_4$ condensation proceeds through the formation of a triphenyltrichloro-methylphosphonium chloride salt and subsequent formation of an acyloxyphosphonium salt. In the presence of an amino acid ester, the nitrogen reacts exclusively at the carbonyl carbon of the acyloxyphosphonium salt to form the dipeptide analog [3].

Synthetic Methods:

L-Phenylalanine methyl ester hydrochloride **3**, L-phenylalanine **4**, and boric acid **5**, were purchased from Aldrich Chemical Company (Milwaukee, WI). Compound **1** was synthesized by the method of Sood *et al.* [1]. Compounds **2** and **6** were synthesized as follows:

Synthesis of sodium N-[(trimethylamineboryl)-carbonyl]-L-phenylalanine (**2**):

N-[(Trimethylamineboryl)-carbonyl]-L-phenylalanine methyl ester **1** (1 g, 3.59 mmol) was suspended in 50 ml distilled water to which 3.6 ml of an 0.986 M aqueous sodium hydroxide

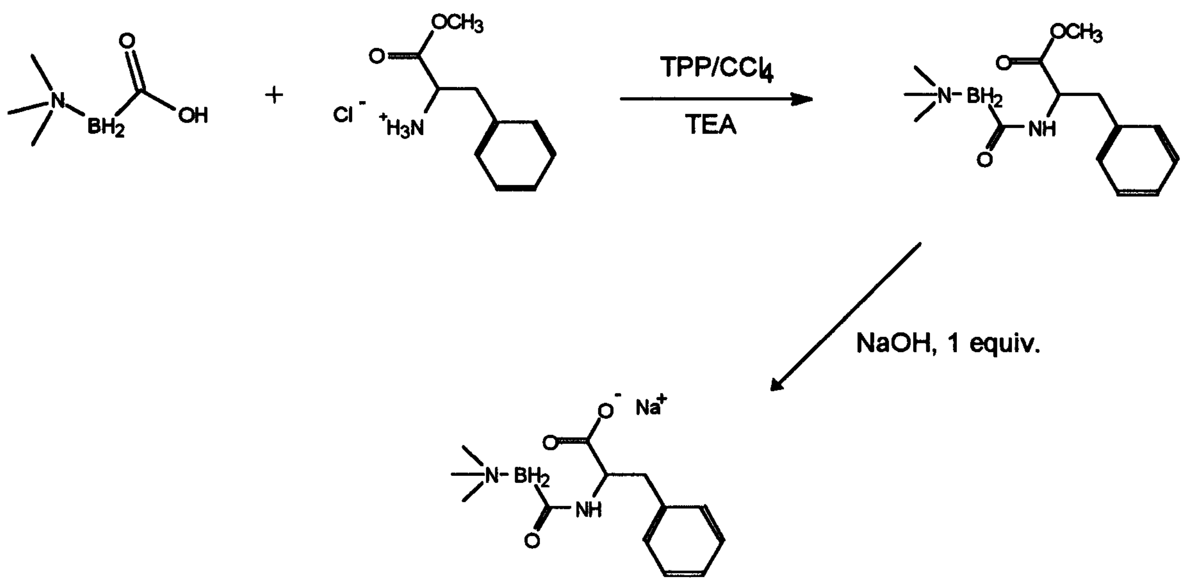

**Scheme I: The Synthesis of Derivatives of the Boronated Dipeptide N-[(Trimethylamineboryl)-carbonyl]-L-phenylalanine Methyl Ester.**

solution (0.14 g, 3.59 mmol) was slowly added while stirring. After 15 minutes, the reaction appeared to be complete by TLC analysis although small amounts of an insoluble white solid are present. The white solid was removed by vacuum filtration and solvent was removed under reduced pressure leaving a white hygroscopic solid. The white hygroscopic solid was further purified by silica gel column chromatography using one void volume of ethyl acetate, followed by ethyl acetate / acetonitrile (1:1) until completed. Combined pure fractions yielded 486.8 mg (47%) of a white hygroscopic solid; $R_f$=0.12 in 1:1 ethyl acetate/acetonitrile. $^1$H-NMR (DMSO-$d_6$): δ 7.16 (m,5H,$C_6H_5$); δ 6.57 (d,1H,NH); δ 4.35 (q,1H,CH); δ 3.00 (ddd,2H,$CH_2$); δ 2.60 (s,9H,$NMe_3$); δ 1.72 (m,2H,$BH_2$); IR ($cm^{-1}$)$_{Nujol}$ : 3450 $\nu_{NH}$, 2380 $\nu_{BH}$, 1575 $\nu_{CONH}$; m.p. 95-97°C (dec.). (Calcd.: C, 54.57%; H, 7.05%; N, 9.79%. Found: C, 54.33%; H, 6.95%; N, 9.57%).

Synthesis of <u>N,N-dimethylglycyl-L-phenylalanine methyl ester</u> (<u>6</u>):

L-Phenylalanine methyl ester hydrochloride (2.09 g, 9.7 mmol) and dimethylglycine were suspended in 30 ml dry tetrahydrofuran (THF) with triethylamine (1.4 ml, 1.01 g, 10 mmol), dicyclohexylcarbodiimide (DCC, 2.24 ml, 2.06 g, 10 mmol) and hydroxybenzotriazole (HOBt, 1.35 g, 10 mmol). The reaction mixture was stirred at room temperature for 48 hours under nitrogen and the solvent was removed by rotary evaporation. The product was purified by silica gel column chromatography using two void volumes of hexane, two void volumes of hexane/ethanol (8:1), and then hexane:ethanol (5:1). The combined fractions of purified product yielded 421.9 mg (17%) of a clear liquid; $R_f$=0.35 in hexane:ethanol (1:1). $^1$H-NMR ($CDCl_3$): δ 7.55 (d,1H,NH); δ 7.26 (m,5H,$C_6H_5$); δ 4.91 (m,1H,CH); δ 3.73 (s,3H,$OCH_3$); δ 3.18 (ddd,2H,$CH_2$); δ 2.92 (d,2H,$CH_2$); δ 2.20 (s,6H,$CH_3$). (Calcd.: C, 63.61%; H, 7.63%; N, 10.60%. Found: C, 63.82%; H, 7.60%; N, 10.57%).

Pharmacological Assays

Compounds **1-6** were tested for cytotoxic activity by homogenizing drugs in a 1 mg/ml solution in 0.05% Tween 80/$H_2O$. These solutions were sterilized by passing them through an acrodisc (45 µ). The following cell lines were maintained by literature techniques [4]: murine $L_{1210}$ lymphoid leukemia, rat UMR 106 osteosarcoma, human $Tmolt_3$ acute lymphoblastic T cell leukemia, HeLa-$S^3$ suspended cervical carcinoma, HeLa solid cervical carcinoma, KB epidermoid nasopharynx, colorectal adenocarcinoma SW480, HCT-8 ileocecal adenocarcinoma, lung bronchogenic MB-9812, A549 lung carcinoma, A431 epidermoid carcinoma, and glioma HS683. Geran *et al.*'s protocol [5] was used to assess the cytotoxicity of the compounds and standards in each cell line. Cell numbers were determined by the trypan blue exclusion technique. Solid tumor cytotoxicity was determined by Liebovitz *et al.'s* method [4] utilizing crystal violet/MeOH and read at 562 nm (Molecular Devices). Values for cytotoxicity were expressed as $ED_{50}$ = µg/ml, i.e. the concentration of the compound inhibiting 50% of cell growth. A value of less than 4 µg/ml was required for significant activity of growth inhibition.

Antihyperlipidemic activity:
Serum cholesterol levels:

$CF_1$ male mice (~28 g) were administered drugs, suspended in 1% carboxymethylcellulose (CMC) at 8 mg/kg/day, I.P., for 16 days. On days 9 and 16, the mice were bled by tail vein bleeding into capillary tubes. Serum was obtained by centrifugation at 3000 x g for 2 min. Total serum cholesterol was determined by the Liebermann-Burchard reaction [6]. Lovastatin (8 mg/kg/day) and clofibrate (150 mg/kg/day) were also tested as standard cholesterol reducing agents.

Serum triglyceride levels:

Serum triglyceride levels were determined by using a commercial kit (triglycerides GPO Trinder 20, Sigma). Lovastatin (8 mg/kg/day) and clofibrate (150 mg/kg/day) were also included as standard triglyceride reducing agents.

Anti-inflammatory activity:
Winter's test:

$CF_1$ male mice (~28 g) were administered experimental drugs, in 0.05% Tween 80 / water, at 8 mg/kg doses, I.P., 3 hours and again at 30 min. prior to injection of 0.05 ml 1% carrageenin, in 0.9% saline, into the plantar surface of the right hind foot. Saline injected into the left hind foot served as a base line control. After 3 hours, both feet were excised at the tibiotarsal (ankle) joints and were weighed according to the modified method of Winter [7]. The control value was 84 mg of edema. The percentage inhibition was determined for the treated group with respect to the control group. Indomethacin (50 mg/kg) and phenylbutazone (10 mg/kg) were included as standard anti-inflammatory agents.

Writhing Reflex:

$CF_1$ male mice were administered experimental drugs at 8 mg/kg, I.P., 20 min. prior to the I.P. administration of 0.5 ml of 1.2% acetic acid according to the methods of Hendershot *et al.* [8] and Vinegar *et al.* [9] After 5 min., the number of stretches, characterized by repeated contractures of the abdominal musculature accompanied by hindlimb extension, was counted over the next 10 min. Control mice demonstrated ~25 stretch reflexes/10 min. Indomethacin (50 mg/kg) was included as a standard analgesic agent.

Protection Against Septic shock:

$CF_1$ male mice (~25g) were administered *Salmonella* lipopolysaccharide (LPS), 10 mg/kg I.P., an amount that is lethal within 48-52 hours [10]. Drugs were administered, at 8 mg/kg, 2 hours before and 2 hours after injection of LPS and every 24 hours afterwards up to 48 hours. Deaths were recorded daily for the length of the study. The percent death at 52 hr. was calculated and compared to the control group, which afforded 16% survival at 52 hr. Indomethacin (50 mg/kg), phenylbutazone (10 mg/kg), pentoxifylline (50 mg/kg), and dexamethasone (1 mg/kg) were included as standard antisepsis agents.

Statistical Analysis:

Data is displayed in tables 1-3 as the means ± standard deviations or standard errors of the mean. N is the number of samples or animals per group. The Student's "t"-test was used to determine the probable level of significance (p) between test samples and control samples.

Results:

Chemistry:

The synthesis of derivatives of the boronated dipeptide N-[(trimethylamine-boryl)carbonyl]-L-phenylalanine methyl ester was successfully accomplished in moderate yields using the coupling agent triphenylphospine/carbon tetrachloride. The structures (Figure I) and purity of the compounds were confirmed using elemental analysis, melting points, and both $^1$H-NMR and infrared spectroscopy. All values were within acceptable limits (data available upon request). N,N-dimethylglycyl-L-phenylalanine methyl ester was synthesized by using dicyclohexylcarbodiimide (DCC) as the coupling agent with low yields, when the standard method was unsuccessful.

*In Vitro* Cytotoxicity:

The compounds demonstrated *in vitro* cytotoxicity primarily in suspended tumor cell lines, e.g., murine $L_{1210}$ lymphoid leukemia, human $Tmolt_3$ T cell acute lymphoblastic leukemia, and HeLa-$S^3$ suspended human uterine cervical carcinoma, with variable activity in human solid tumor cell cultures (Table 1). Compounds **2**, **3**, and **6** demonstrated $ED_{50}$ values against $L_{1210}$ growth < 3 μg/ml. Rat osteogenic sarcoma UMR-106 activity was noted for only compound **4** ($ED_{50}$ < 4 μg/ml). Compound **1** produced the best activity in the $Tmolt_3$ screen with an $ED_{50}$ value of 1.31 μg/ml. Compounds **2-6** afforded $ED_{50}$ values < 3 μg/ml in the HeLa-$S^3$ uterine carcinoma screen. In contrast only compounds **1** and **2** were active against solid HeLa cervical carcinoma growth. Against KB nasopharynx growth, compound **1** alone was active ($ED_{50}$ = 1.99 μg/ml). Against MB9812 bronchogenic lung growth, only compound **2** was active ($ED_{50}$ = 1.33 μg/ml). Compound **2** alone inhibited the growth of lung carcinoma A549 with an $ED_{50}$ of 2.97 μg/ml. None of the compounds tested were active against the growth of colorectal adenocarcinoma SW-480, ileum HCT-8, skin epidermoid A431, or HS-683 glioma.

*In Vivo* Hypolipidemic Activity:

Compounds **1-3**, and **6** demonstrated significant activity as hypolipidemic agents on both days 9 and 16, at 8 mg/kg/day, I.P., in $CF_1$ male mice (Table II). The compounds were similarly effective, reducing serum cholesterol levels in $CF_1$ male mice 30-50%, on day 16. The most effective hypocholesterolemic agent was compound **1**, which caused a 48% reduction in serum cholesterol levels on day 16. L-Phenylalanine methyl ester, **3**, which reduced serum cholesterol levels 39%, on day 16, had similar efficacy as the other boron containing agents. L-phenylalanine, **4**, and boric acid, **5**, did not significantly reduce serum cholesterol levels over 16 days.

Serum triglycerides were only significantly reduced by compounds **1**, **3**, and **6** on day 16 (Table II). The L-phenylalanine methyl ester, **3**, was approximately equally active as the parent compound **1**. The other metabolites were not as active in reducing serum triglycerides at 8 mg/kg/day.

Winter's Test:

The ability to significantly reduce the carageenan induced inflammation in $CF_1$ male mice footpads was demonstrated by compounds **1**, **2** and **6** (Table III). Compounds **3-5** did not appear to possess any activity. Compound **2** demonstrated the best inhibition of 40%. N,N-dimethylglycyl-L-phenylalanine methyl ester, **6**, was more active than the boronated parent compound, **1** with 32% inhibition compared to 23% inhibition at 8 mg/kg x 2 for the parent **1**.

Writhing Assay:

All of the compounds tested demonstrated the ability to significantly block local pain (Table III). The most active compound from the derivatives of the boronated dipeptide N-[(trimethylamine-boryl)-carbonyl]-L-phenylalanine methyl ester was **6**, which reduced mouse writhing reflex 52%, followed closely by **3**, which provided a 50% reduction in writhing reflex. Both were significantly more active than the parent compound, **1** with 17% reduction at 8 mg/kg.

**Table I: *In Vitro* Cytotoxicity of Derivatives of the Boronated Dipeptide N-[(Trimethylamineboryl)carbonyl]-L-phenylalanine Methyl Ester**

| | $ED_{50}$ value (μg/ml)* | | | | | | | | | | | |
|---|---|---|---|---|---|---|---|---|---|---|---|---|
| (N=6) | Murine | Rat | Human | | | | | | | | | |
| Cmpd | $L_{1210}$ | UMR-106 | $Tmolt_3$ | HeLa-$S^3$ | HeLa solid | KB | SW480 colon | HCT-8 ileum | MB-9812 lung | A549 lung | A431 skin | HS-683 glioma |
| 1 | 2.07 | 6.81 | 3.03 | 2.37 | 6.61 | 5.12 | 8.07 | 8.74 | 9.53 | 9.69 | 4.82 | 12.4 |
| 2 | 4.66 | 3.26 | 5.13 | 2.92 | 7.86 | 8.25 | 4.78 | 7.08 | 8.19 | 9.60 | 9.85 | 9.99 |
| 3 | 3.08 | 4.22 | 6.87 | 2.92 | 8.83 | 8.51 | 6.13 | 9.09 | 6.85 | 8.03 | 9.03 | 10.4 |
| 4 | 2.13 | 8.90 | 3.38 | 2.47 | 9.13 | 7.11 | 7.89 | 7.61 | 5.36 | 7.62 | 7.69 | 7.29 |
| 5 | 3.34 | 5.96 | 1.31 | 4.01 | 3.38 | 1.99 | 8.19 | 8.13 | 5.78 | 10.82 | 4.20 | 6.88 |
| 6 | 2.68 | 4.00 | 4.22 | 2.37 | 1.82 | 8.02 | 4.29 | 5.13 | 1.33 | 2.97 | 5.83 | 14.2 |
| standard agents for comparison | | | | | | | | | | | | |
| 6-MP | 2.43 | 9.13 | 1.62 | 2.12 | 5.61 | 11.04 | 3.61 | 1.15 | 4.29 | 4.71 | 3.42 | 4.46 |
| Ara-C | 2.43 | 0.86 | 2.67 | 2.13 | 4.74 | 2.84 | 3.42 | 2.54 | 6.16 | 6.28 | 0.92 | 1.88 |
| Hydroxyurea | 2.67 | 2.87 | 4.47 | 1.96 | 8.12 | 5.27 | 7.33 | 1.77 | 7.18 | 8.89 | 3.21 | 2.27 |
| 5-FU | 1.41 | 3.52 | 2.14 | 2.47 | 4.11 | 1.25 | 3.09 | 1.12 | 5.64 | 3.58 | 0.61 | 1.28 |
| VP-16 | 1.83 | 3.57 | -- | 7.87 | 3.05 | 3.32 | 3.34 | 3.78 | 3.50 | 4.74 | 0.71 | 2.44 |

* Significant $ED_{50}$ values were less than 4 μg/ml.

**Table II: *In Vivo* Hypolipidemic Activity of Derivatives of the Boronated Dipeptide N-[(Trimethylamine-boryl)carbonyl]-L-phenylalanine Methyl Ester in $CF_1$ Male Mice at 8 mg/kg/day, I.P. for 16 Days.**

Percent of Control (mean ± s.d.)

| (N = 6) | Serum Cholesterol | | Serum Triglycerides |
|---|---|---|---|
| | Day 9 | Day 16 | Day 16 |
| Control[a] | 100±5[b] | 100±5[c] | 100±4[d] |
| 1[e] | 75±6* | 52±3* | 67±5* |
| 2 | 71±5* | 61±4* | 88±5 |
| 3 | 69±5* | 61±5* | 63±5* |
| 4 | 90±5 | 90±6 | 94±6 |
| 5 | 96±7 | 100±5 | 101±6 |
| 6 | 76±4* | 74±5* | 80±5* |
| Lovastatin[f] | 85±4 | 82±5* | 86±7 |
| Clofibrate[g] | 87±6 | 78±6* | 75±6* |

* $p \leq 0.001$

[a] 1% carboxymethylcellulose [b] 125 mg/dL serum cholesterol [c] 127 mg/dL serum cholesterol

[d] 137 mg/dL serum triglyceride [e] as reported by Sood *et al.* [1] [f] 8 mg/kg/day [g] 150 mg/kg/day

Septic Shock:

All the compounds tested provided protection against LPS induced septic shock (Table III). Compound **6**, at 8 mg/kg/day, demonstrated the best protection against septic shock with 100% of the mice surviving. Compound **6** was significantly more active than the parent compound, **1** which demonstrated only 50 % protection at 8 mg/kg/day. After treatment with compounds **2-4** mouse survival was 83% after 48 hours, while treatment with compound **5** resulted in 67% mouse survival after 48 hours. All of the proposed metabolites of compound **1**, compounds **3-6**, were more effective than the parent compound.

**Table III: *In Vivo* Anti-inflammatory, Analgesic, and Sepsis Prophylactic Activities of Derivatives of the Boronated Dipeptide N-[(Trimethylamine-boryl)carbonyl]-L-phenylalanine Methyl Ester in $CF_1$ Male Mice at 8 mg/kg, I.P.**

| (N = 6) Compound # | Winter's Test % Control[a] | Writhing % Control[b] | Septic Shock % Protection |
|---|---|---|---|
| Control | 100±11 | 100 | 17 |
| 1[c] | 77±5* | 83 | 50 |
| 2 | 60±6* | 97 | 83 |
| 3 | 90±4 | 50 | 83 |
| 4 | 83±5 | 77 | 83 |
| 5 | 80±5 | 95 | 67 |
| 6 | 68±7* | 48 | 100 |
| Indomethacin[d] | 22±4* | 43 | 33 |
| Phenylbutazone[e] | 53±4* | -- | 0 |
| Pentoxifylline[f] | -- | -- | 66 |
| Dexamethasone[g] | -- | -- | 83 |

* $p \leq 0.001$

[a] 84 mg increase in paw weight [b] 25 stretch reflexes per 10 min. [c] as reported by Sood *et al.* [1]

[d] 50 mg/kg [e] 10 mg/kg [f] 50 mg/kg [g] 1 mg/kg

Discussion:

This investigation of the proposed metabolites of N-[(trimethylamineboryl)-carbonyl]-L-phenylalanine methyl ester **1**, suggests that some of the metabolites may be responsible for the observed pharmacological action. For example in the human lung A549 and MB9812 tumor screens, the metabolite N-[(trimethylamineboryl)-carbonyl]-L-phenylalanine **2** afforded better cytotoxic activity than the parent compound **1**. In the $L_{1210}$ murine lymphoid leukemia screen **2**, **3**, and **6** demonstrated better activity than the parent **1**. In the HeLa-S3 screen the metabolites were active but the parent was not. Whereas in the human $Tmolt_3$ T cell leukemia screen only the parent **1** was significantly active. In the HeLa and KB nasopharynx all of the metabolites were inactive but the parent was active. Only L-phenylalanine **4** was marginally active in the rat UMR-106 osteosarcoma screen. In some of the tumor lines (e.g., colon, ileum, skin, and glioma) none of the compounds demonstrated cytotoxicity. It should be pointed out that these are in vitro studies and it is unknown what the capability of each tumor line had with regard to metabolizing compound **1**. One would assume that hydrolytic enzymes (esterases and amidases) were present at some concentration in all cell lines. There may also be species differences between murine, rat, and human cell lines.

In the hypolipidemic *in vivo* screen, compounds **2**, **3**, and **6** demonstrated moderate activity of 39%, 39%, and 26%, respectively. However, only compound **3** was as active as the parent **1** with a 37% reduction in serum triglyceride levels at 8 mg/kg/day. This suggested that the boron atom was not necessary for hypolipidemic activity.

In the Winter's test compounds **2** and **6** (methyl ester derivatives) demonstrated slightly improved activity (32% and 40% respectively) over the parent **1** (23%) at 8 mg/kg. In the writhing / local pain assay compounds **3** and **6** demonstrated significantly improved activity (50-52% reduction) over the parent **1** (17% reduction). In the LPS endotoxic shock assay the parent **1** only resulted 50% protection from death. Compounds **2**, **3**, and **4** demonstrated improved protection with 83% and compound **6** afforded 100% protection suggesting that all of the metabolites with and without the methyl ester or boron were more effective than N-[(trimethylamineboryl)-carbonyl]-L-phenylalanine methyl ester **1**. Thus it may be concluded that the metabolites of **1** were responsible for some of the observed pharmacological activity of compound **1**. This response varied in mice with the type of pharmacological activity tested.

References:

1 Sood, A., Sood, C.K., Spielvogel, B.F., Hall, I.H., *Eur. J. Med. Chem.*, **1990**, 25, 301-308.
2 Miller,III, M.C., Shrewsbury, R.P., Elkins, A.L., Sood, A., Spielvogel, B.F., Hall, I.H., *J. Pharm. Sci.*, **1995**, 84, 933-936.
3 Lee, J., *J. Am. Chem. Soc.*, **1966**, 88, 3440-3441.
4 Leibovitz, A., Stinson, J.C., McCombs, III, W.B., McCoy, C.E., Mazur, K.C., Mabry, N.D., *Cancer Res.*, **1976**, 36, 4562-4569.
5 Geran, R.I., Greengerg, N.H., MacDonald, M.M., Schumacher, A.M., Abbott, B.J., *Cancer Chem. Rep.*, **1972**, 3, 7-9.
6 Ness, A.T., Pastewka, J.V., Peacock, A.C., *Clin. Chim. Acta.*, **1964**, 10, 229-237.
7 Roszkowski, A.P., Rooks, W.H., Tomolonis, A., Miller, L.M., *J. Pharmacol. Exp. Ther.*, **1971**, 179, 114-123.
8 Hendershot, L.C., Forsaith, J., *J. Pharmcol. Exp. Ther.*, **1959**, 125: 237-240.
9 Vinegar, R., Truax, J.F., Selph, J.L., *Eur. J. Pharmacol.*, **1976**, 37: 23.
10 Noronha-Blob, L., Lowe, V.C., Weitzberg, M., Burch, R.M., *Eur. J. Pharmacol.*, **1991**, 199, 387-388.

**Received: September 10, 1996 - Accepted: September 17, 1996 - Received in revised camera-ready format: September 24, 1996**

